# Monitoring changes in circulating tumour cells as a prognostic indicator of overall survival and treatment response in patients with metastatic melanoma

**DOI:** 10.1186/1471-2407-14-423

**Published:** 2014-06-11

**Authors:** Dragana Klinac, Elin S Gray, James B Freeman, Anna Reid, Samantha Bowyer, Michael Millward, Melanie Ziman

**Affiliations:** 1 School of Medical Sciences, Edith Cowan University (ECU), 270 Joondalup Drive, Joondalup, Perth, WA 6027, Australia; 2 Department of Medical Oncology, Sir Charles Gairdner Hospital, Nedlands, WA, Australia; 3 School of Medicine and Pharmacology, University of Western Australia, Crawley, WA, Australia; 4 School of Pathology and Laboratory Medicine, University of Western Australia, Crawley, WA, Australia

**Keywords:** Circulating tumour cells, Melanoma, Vemurafenib

## Abstract

**Background:**

New effective treatments for metastatic melanoma greatly improve survival in a proportion of patients. However biomarkers to identify patients that are more likely to benefit from a particular treatment are needed. We previously reported on a multimarker approach for the detection of heterogenous melanoma circulating tumour cells (CTCs). Here we evaluated the prognostic value of this multimarker quantification of CTCs and investigated whether changes in CTC levels during therapy can be used as a biomarker of treatment response and survival outcomes.

**Methods:**

CTCs were captured by targeting the melanoma associated markers MCSP and MCAM as well as the melanoma stem cell markers ABCB5 and CD271. CTCs were quantified in 27 metastatic melanoma patients treated by surgery or with vemurafenib, ipilimumab or dacarbazine. Patients were enrolled prospectively and CTC counts performed at baseline (prior to treatment), during and after treatment.

**Results:**

Baseline CTC numbers were not found to be prognostic of overall survival nor of progression free survival. However, a low baseline CTC number was associated with a rapid response to vemurafenib therapy. A decrease in CTCs after treatment initiation was associated with response to treatment and prolonged overall survival in vemurafenib treated patients.

**Conclusions:**

Measuring changes in CTC numbers during treatment is useful for monitoring therapy response in melanoma patients and for providing prognostic information relating to overall survival. Further studies with larger sample sizes are required to confirm the utility of CTC quantification as a companion diagnostic for metastatic melanoma treatment.

## Background

The prognosis for patients with metastatic melanoma has improved significantly over the last three years with the implementation of novel targeted therapeutic agents and immunotherapies. However targeted therapies develop drug resistance within 12 months [1,2] and immunotherapies are only effective in a small proportion of patients [3]. Early prediction of treatment failure and the ability to detect recurrence after treatment would allow patients who fail on one therapy to be switched early to different modalities, reducing disease progression and the cost of a futile therapy.

The presence of circulating tumour cells (CTCs) has been identified as an independent prognostic marker in a number of metastatic cancers [4-9]. The number of CTCs prior to initiation, during and after therapy has been shown to be indicative of the length of progression free survival (PFS) and of overall survival (OS) [4,10,11]. Temporal monitoring of CTC numbers during and after therapy showed that a decrease in CTCs correlated reasonably well with the clinical course of disease and also appears useful for evaluating the patient’s response to therapy [4,8,12,13]. Moreover the predictive value for survival based on CTC enumeration has been shown to be superior to standard monitoring tests such as prostate-specific antigen (PSA) in castration-resistant prostate cancer [4] and tumour imaging in metastatic breast cancer [14]. While most clinical studies, so far, have focused on CTC enumeration in guiding prognosis in metastatic cancer patients, current research is exploring the pharmacodynamic and predictive biomarker utility of CTCs [15].

For melanoma, relatively few studies have detailed the prognostic value of CTCs. Two studies have shown that the number of CTCs is prognostic of OS, with more than 2 CTCs per 7.5 ml of blood associated with shorter survival [6,16]. These two studies made use of the CellSearch Melanoma Kit which captures melanoma cell adhesion molecule (MCAM)-expressing cells and detects melanoma chondroitin sulfate proteoglycan (MCSP)-positive cells as CTCs [16]. However, melanoma tumours have highly heterogeneous expression patterns [17] and it is likely their derived CTCs also exhibit this heterogeneity. Thus in a previous study we undertook a novel strategy by targeting a combination of melanoma associated antigens, MCSP and MCAM and previously described stem-cell markers, ATP-binding cassette sub-family B member (ABCB5) [18] and CD271 [19], to enrich CTCs. This approach allowed for a more efficient capture of heterogeneous melanoma CTCs relative to targeting a single marker [20]. Using this multimarker approach, we previously demonstrated that patients at later clinical disease stages have significantly greater numbers of CTCs than those at earlier stages [20]. In the present study we use our multimarker immunomagnetic enrichment method to evaluate the prognostic value of detecting heterogeneous CTCs and to investigate whether changes in CTC levels during therapy correlate with survival outcomes as well as treatment response as measured by radiographic Response Evaluation Criteria in Solid Tumours (RECIST), version 1.1 [21].

## Methods

### Study design

A prospective study was conducted at the Sir Charles Gardner Hospital (SCGH), Perth, Western Australia. Patients were enrolled in the study prior to treatment initiation. Treatment included surgery, standard chemotherapy with dacarbazine, targeted agents including BRAFV600E inhibitors either alone (vemurafenib) or in combination with a MEK inhibitor (dabrafenib/trametinib), as well as immunotherapy (ipilimumab). Written informed consent was obtained from all patients. The study was approved by the Human Research Ethics Committees of Edith Cowan University (No. 2932) and Sir Charles Gairdner Hospital (No. 2007-123).

### Patient follow up

Patients underwent baseline assessment of medical history, physical examination, and radiographic tumour assessment with computer tomography (CT) or positron emission tomography (PET) scan. Patients were treated at the discretion of their treating oncologist as appropriate for their disease stage, mutational status and performance status. Patients underwent clinical assessment at least monthly, including a physical examination and assessment of biochemical parameters. Tumour responses were assessed radiologically at two to three monthly intervals. CT scans were assessed by RECIST 1.1 criteria and classified as having a complete response (CR), partial response (PR), stable disease (SD) or progressive disease (PD).

### CTC enumeration

CTC counts were performed at baseline, before the initiation of therapy, and throughout therapy. Patient peripheral blood samples were collected in 4 ml EDTA tubes, stored at 4°C, and processed within 24 hours of collection. CTCs were enriched and enumerated as previously described [20]. In summary, whole blood was treated with red blood cell lysing buffer and remaining cells were incubated with immunomagnetic beads coated with antibodies against MCSP, MCAM, ABCB5 and CD271 cell surface antigens to target CTCs. The resulting CTC enriched fraction was washed to remove non-specifically bound leukocytes, fixed with 4% paraformaldehyde and stained with anti-CD45 antibody, followed by an AF488 conjugated secondary antibody (Abcam, Cambridge, MA) and mounted with media containing DAPI for nuclear staining. Cells were quantified by microscopy where CTCs were defined as bead bound cells with a DAPI stained nucleus that were negative for CD45 expression.

### Statistics

Association of baseline CTC number and individual clinical, biochemical and genetic factors were compared using *χ*^2^ test. PFS time was calculated from baseline date to the date of first reported PD. OS time was calculated from baseline date to the date of death. Response time was calculated from the date at baseline to the date of first reported PR or CR. CTC number at baseline or the change in CTC number after commencement of treatment, was subject to univariate Cox proportional hazards regression analysis for association with PFS, OS and response to treatment. Results were analysed in SPSS 21.0 and GraphPad Prism 5.

## Results

### Patient demographics

A total of 27 patients with metastatic cutaneous melanoma were enrolled in the study between September 2011 and January 2013 (Table 1). At the time of analysis, November 2013, 20 (74%) had experienced disease progression and 12 (44%) had died, resulting in a median PFS time of 32 weeks (8 months) and a median OS time of 53 weeks (12 months). The average length of follow up of patients was 53 weeks (range, 5-117 weeks).

**Table 1:** Baseline demographic and clinical characteristics of patients

**Characteristic**	**Number**	
**Number of patients**	**N = 27**	**%**
Age at enrolment (years)		
Median	59	
Range	23-80	
Gender		
	Male	15	56%
Female	12	44%	
Stage of disease at baseline			
Stage IV			
M1a	4	15%	
M1b	4	15%	
M1c	19	70%	
Period of time with metastases			
Median	2.5 months		
Range	0-115 months		
LDH			
Normal (≤333)	14	54%	
High (>333)	4	15%	
Unknown	9	35%	
BRAF mutation			
WT	9	35%	
V600E	11	42%	
V600K	5	19%	
K601E	1	4%	
unknown	1	4%	
Treatment (post enrolment)			
Vemurafenib	12	46%	
Ipilimumab	4	15%	
Dacarbazine	5	19%	
Surgery	4	15%	
Dabrafenib/Trametinib	1	4%	
No treatment	1	4%	

### Analysis of baseline CTC enumeration

Of the 27 patients enrolled in the study, 22 were sampled at baseline, prior to treatment. For the other 5 individuals, blood collection started after commencement of treatment, and they were therefore not included in the baseline CTC analysis.

We have previously reported that one cell defined as a CTC may on occasion be found in 4 ml of blood from healthy individuals; hence here we included only those patients with 2 or more CTCs in 4 ml of blood. We found a median of 4 (range 0-10) CTCs at baseline, with 17 patients (72%) presenting with 2 or more CTCs.

**Figure 1:**
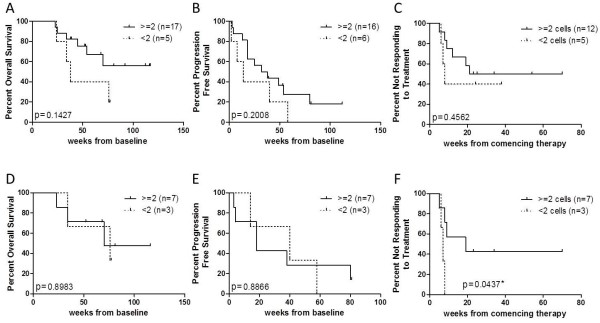
**Kaplan-Meier curves for (A, D) OS, (B, E) PFS and (C, F) time to response of all melanoma patients with > =2 cells and <2 CTCs in 4.5 ml of blood. (C)** Only patients undergoing system therapies were analysed for treatment response, N = 17. Patients treated with the B-Raf inhibitor vemurafenib were analysed separately **(D, E and F)**.

A Kaplan-Meier analysis was performed to determine the association between baseline CTCs and prognostic values such as OS, PFS and response to treatment (Figure 1A, B and C). Log-rank test did not show an association between the number of baseline CTCs and any of these three outcome measurements. The analysis was performed repeatedly using different cut off values to define a favourable or unfavourable CTC number, at 3, 4 or 5 CTCs, but no statistical significance was found in any of these comparisons.

The study participants had undergone a variety of treatments, which have different response rates and mechanisms of action. These different treatments may have distinct effects on disease progression therefore altering the predictive value of baseline CTC numbers. Thus, we performed further analyses focusing on the vemurafenib treated patients only, given that they were a substantial group of the study subjects for which a baseline CTC count was obtained, 10 of 22 (45%). Once again, no predictive value was found between baseline CTCs and OS or PFS in this subgroup (Figure 1D and E). However, we found that vemurafenib treated patients with detectable CTCs (≥2) at baseline took longer to respond to treatment than those with <2 CTCs (HR 0.11, 95% CI 0.012-0.93, log-rank P = 0.0437) (Figure 1F). As above, the same analysis was performed for different cut-off values with a 2 CTC cut-off showing the best predictive value.

### Changes in CTCs as predictive of OS and response to treatment

Next we evaluated whether changes in the number of detected CTCs after treatment initiation is predictive of patient response to treatment and disease progression. We collated CTC counts during the first 12 weeks after treatment initiation in 13 out of the 22 patients with baseline counts. Of those, 8 were treated with vemurafenib, 3 with ipilimumab and 2 with dacarbazine. The slope of a linear regression curve was calculated for each patient, including at least three time points and two CTC counts per time point. The slope of the curve was used as an indicator of CTC changes during this period; with a positive slope indicating an increase or no change in CTC numbers and a negative slope indicating a decrease in CTCs.

**Figure 2:**
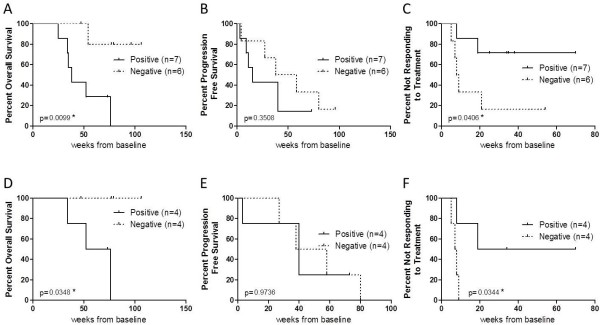
**Prognostic significance of CTC number.** Kaplan-Meier curves for **(A, D)** OS, **(B, E)** PFS and **(C, F)** time to response of all melanoma patients with a positive and negative change in CTC number after treatment, calculated as the slope of plotted CTC counts over the first 12 weeks. Patients treated with the B-Raf inhibitor vemurafenib were analysed separately **(D, E and F)**.

**Figure 3:**
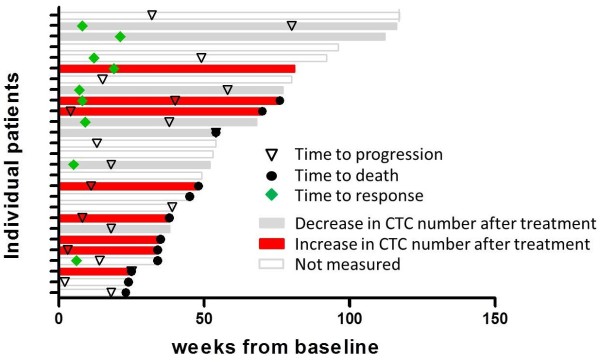
**Times to response, progression and death among the 27 patients in the study.** For patients where the changes in CTC numbers were evaluated, the bars were coloured red for an increase or grey for a decrease.

**Figure 4:**
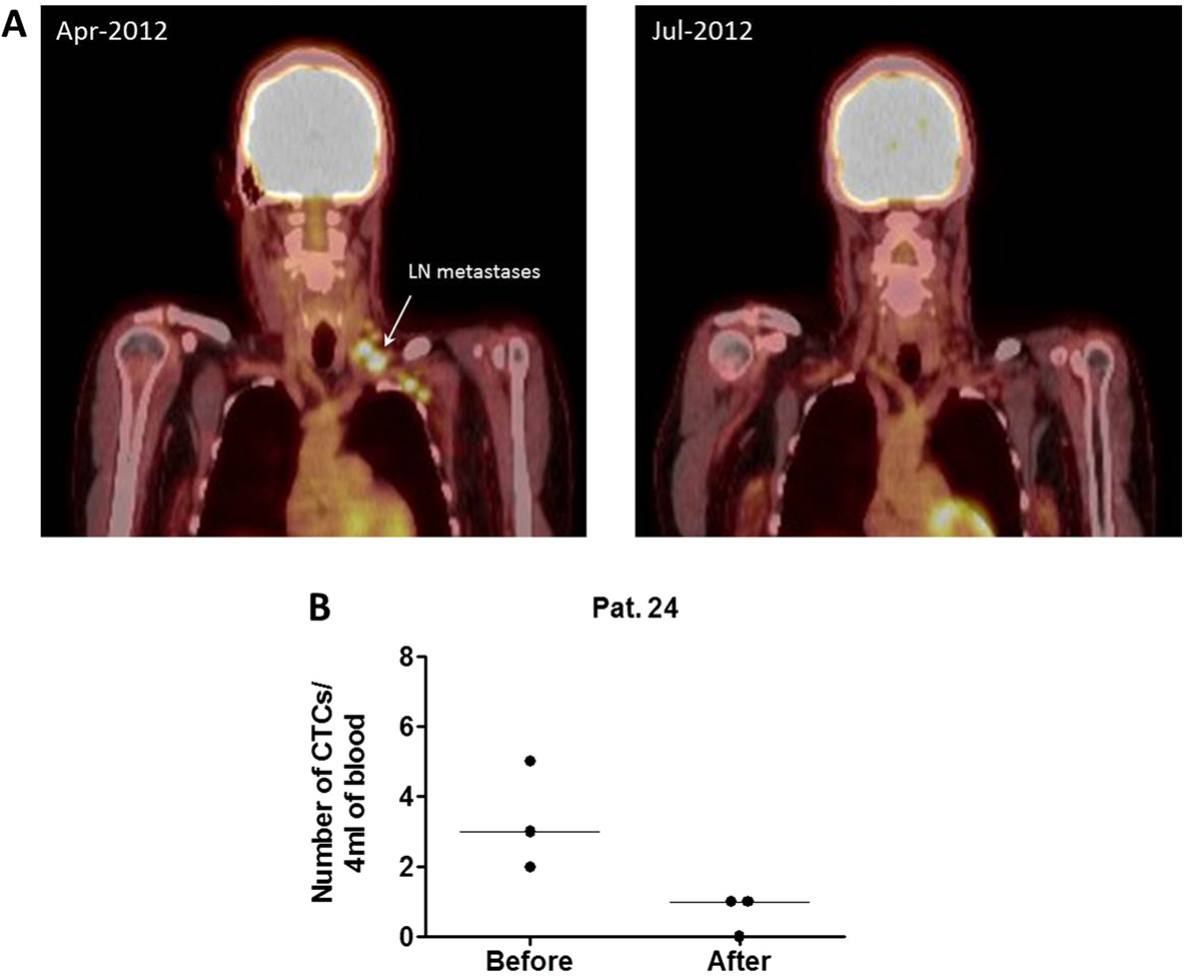
**Therapy response in a metastatic melanoma patient treated with vemurafenib. (A)** Representative images of the PET scans before and during vemurafenib treatment. The arrow indicates lymph node metastasis detected prior to treatment and a complete metabolic response 2 months after treatment. **(B)** Reduction in the number of CTCs in 4 ml of whole blood in the same patient. A total of 12 ml of blood was collected at each time point, 3 × 4 ml tubes. The graph illustrates the number of CTCs found in each of the three blood samples and the median for each time point.

Log-rank Mantel-Cox tests (Figure 2A, B and C) demonstrated that a decrease in CTCs after treatment (negative slope) is associated with longer OS (HR 7.7, 95% CI 1.6-36.8, log-rank P = 0.0099) and shorter time to respond to treatment (HR 0.19, 95% CI 0.04-0.93, log-rank P = 0.0406). No association was observed between changes in CTCs and PFS (P = 0.3508). A sub-analysis of only vemurafenib treated patients produced similar results (Figure 2D, E and F). A decrease in CTCs in patients treated with vemurafenib was associated with longer OS (HR 12.7, 95% CI 1.2-135.5, log-rank P = 0.0348). Of note, none of the vemurafenib treated patients with a decrease in CTCs died during the follow up period (Figure 3). Moreover, patients with a decrease in CTCs had a faster response to treatment (HR 0.12, 95% CI 0.02-0.86, log-rank P = 0.0344). All vemurafenib treated patients with a decrease in CTCs had a documented objective response within the first 12 weeks after treatment. Data from a representative patient is shown in Figure 4, illustrating the concomitant reduction in metastatic growth and the number of CTCs after 2 months of treatment with vemurafenib.

## Discussion

The detection of two or more CTCs in 7.5 ml of blood from metastatic melanoma patients using the CellSearch system has been found to be prognostic of overall survival [6,16]. The CellSearch melanoma kit identifies MCAM/MCSP double positive, CD45/CD34 negative cells as CTCs. For our study, we implemented a multi-marker antibody panel for CTC enrichment which, as previously demonstrated, results in detection of more CTCs than when a single marker is targeted [20]. Using this multi-marker approach we show here that a decrease in CTCs after therapy initiation is associated with response to treatment and prolonged OS in vemurafenib treated patients.

In our study, baseline CTC number (prior to treatment) was not prognostic of OS nor of disease free survival, which contrasts with results reported by Khoja et al. [6]. The frequency of patients with 2 or more CTCs was similar between studies with around 1 in 4 patients defined as CTC positive. Although our sample size was relatively small and perhaps not sufficiently powered to detect limited difference in frequencies, we did not detect even a trend towards an association between the presence of CTCs and OS. We observed that our patients in general had a longer OS with a median of 53 weeks (12 months), compared to a maximum of 7.2 months for the ≥2 CTCs group reported by Khoja et al. It is possible that this discrepancy might be because 62% our patients were treated with more effective therapies (BRAF inhibitors and ipilimumab) compared to 27% in their study.

Interestingly, we observed that patients treated with vemurafenib with <2 CTCs at baseline rapidly responded to treatment. However it is unclear why this rapid treatment response did not translate into a longer PFS and OS. In the BRIM3 study, vemurafenib was effective at suppressing disease progression leading to death in the early phase (on average 97 days), however, after a short period this effect ended and patients reverted to the pattern of mortality risk observed in individuals treated with dacarbazine [2]. Furthermore, Sosman et al. observed that although most responses to vemurafenib are rapid, a proportion of patients (11%) had a delayed response more than 6 months later accompanied by longer PFS and OS [22]. This contrasting effect where delayed responses result in longer survival may explain why we did not observe an association with OS despite the association between low baseline CTC count and rapid response to vemurafenib.

A key finding in our study is the relationship between changes in CTCs during treatment and patient OS. We observed a decrease in CTC numbers in 46% of patients following initiation of treatment and this reduction was strongly associated with survival time (HR 7.7, CI 1.6-36.8, log-rank P = 0.0099). This association was still significant when only vemurafenib treated patients were analysed (P = 0.0348). To a lesser extent, a decrease in CTCs was also associated with response to treatment (P = 0.0406), predominantly in the vemurafenib group (P = 0.0344).

This is the first time that changes in CTC number have been shown to be prognostic of OS and treatment response in melanoma patients and provides initial data to support larger studies to evaluate the prognostic value of CTCs and the effect of different therapies on the number of CTCs in patients with metastatic melanoma.

An added benefit of our method is that it enriched for a variety of melanoma CTCs; while it targets those detected by the CellSearch Kit (MCSP and MCAM positive), it also targets CTCs expressing melanoma initiating cell markers (ABCB5 and CD271), which might be excluded by other methods. Further studies on the dynamics of each of these cell types, particularly in response to treatment are important and currently underway in our laboratory.

## Conclusion

Measuring pharmacodynamic changes in CTC numbers during treatment is useful for monitoring therapy response in melanoma patients and for providing prognostic information relating to OS. Further studies with larger sample sizes are required to confirm these observations.

## Abbreviations

ABCB5: ATP-binding cassette sub-family B member 5; CTCs: Circulating tumour cells; MCAM: Melanoma cell adhesion molecule; MCSP: Melanoma chondroitin sulfate proteoglycan; RECIST: Response evaluation criteria in solid tumours; CT: Computerised tomography; PET: Positron emission tomography; CR: Complete response; PR: Partial response; SD: Stable disease; PD: Progressive disease; PFS: Progression free survival; OS: Overall survival.

## Competing interests

The authors declare that they have no competing interests.

## Authors’ contributions

DK designed the study, quantified CTCs, recorded the data and performed some statistical analyses. ESG performed statistical analyses and wrote the manuscript. JBF quantified CTCs and drafted the manuscript. SB and AR coordinated sample collection and patient clinical data. MM recruited participants and provided patient blood samples. MZ conceived of the study, participated in its design and coordination and critically revised the manuscript. All authors read and approved the final manuscript.

## Authors’ information

Dragana Klinac and Elin S Gray are joint first authors.

## Pre-publication history

The pre-publication history for this paper can be accessed here:

http://www.biomedcentral.com/1471-2407/14/423/prepub
